# Comparison of 5.0T and 3.0T time-of-flight MR angiography for visualizing the anterior choroidal artery

**DOI:** 10.1186/s12883-026-05019-9

**Published:** 2026-06-12

**Authors:** Zhangzhu Li, Mingyan Shang, Min Fu, Zhensong Wang, Liang Yin, Dan Yu, Jie Gan

**Affiliations:** 1 Department of Radiology, Shandong Provincial Third Hospital, Shandong University, Jinan, China; 2 United Imaging Research Institute of Intelligent Imaging, Beijing, China

**Keywords:** Magnetic resonance angiography, Anterior choroidal artery, Cerebral arteries, Image quality

## Abstract

**Background:**

This study aimed to compare the image quality of 5.0T and 3.0T time-of-flight (TOF) magnetic resonance angiography (MRA) for visualization of the anterior choroidal artery (AChA).

**Methods:**

This retrospective study included patients who underwent both 3.0T and 5.0T TOF-MRA within a 48-hour interval. Objective image quality was assessed using signal-to-noise ratio (SNR) and contrast-to-noise ratio (CNR). Subjective image quality was independently evaluated by two radiologists using segment-based AChA visibility scores, and quantitative measurements of vessel length and diameter were obtained.

**Results:**

A total of 42 patients who underwent both 3.0T and 5.0T TOF-MRA were included in the paired analysis. Compared with 3.0T imaging, 5.0T TOF-MRA demonstrated significantly higher objective image quality, with increased signal-to-noise ratio (93 ± 5 vs. 65 ± 3) and contrast-to-noise ratio (75 ± 6 vs. 52 ± 4) (both *P* < 0.001). Subjective analysis showed significantly higher visibility scores for distal anterior choroidal artery segments on 5.0T imaging, particularly for the A2 (*P* = 0.007) and A3 (*P* < 0.001) segments. Paired morphological measurements further demonstrated greater apparent AChA length (left: 24.4 ± 7.3 mm vs. 18.7 ± 9.2 mm; *P* < 0.001) and diameter (left: 1.1 mm (1.0-1.2) vs. 0.9 mm (0.7–1.1); *P* < 0.001) on 5.0T TOF-MRA compared with 3.0T imaging.

**Conclusions:**

5.0T TOF-MRA improved visualization of the AChA, particularly its distal segments, compared with 3.0T imaging; further studies are needed to clarify its clinical implications.

**Clinical trial number:**

Not applicable.

**Supplementary Information:**

The online version contains supplementary material available at 10.1186/s12883-026-05019-9.

## Background

The anterior choroidal artery (AChA) is a critical component of the cerebral vascular network. Despite its small size, it plays a vital role by supplying blood to essential motor and sensory pathways, including the posterior limb of the internal capsule, the thalamocortical tract, and the visual and auditory radiations [1]. AChA syndrome, resulting from ischemic damage, often presents with a range of severe neurological symptoms, including contralateral hemiplegia, hemianesthesia, and hemianopsia, and can even affect higher cognitive functions [2, 3]. Clinical case studies frequently report on this “triple syndrome,” underscoring the devastating impact of even a small infarct in this territory [4, 5]. While Digital Subtraction Angiography (DSA) is considered the gold standard with a high display rate for the AChA (92.2%) [6], its invasive nature poses inherent risks, limiting its application for routine screening and widespread clinical use [7]. This underscores the need for a reliable, high-resolution, non-invasive imaging alternative.

Although parenchymal imaging with diffusion-weighted imaging and fluid-attenuated inversion recovery (FLAIR) can usually confirm an established AChA territory infarct, direct visualization of this artery remains clinically important in several settings [8]. Detailed delineation of the AChA and its perforating branches is crucial for preoperative risk assessment in aneurysm clipping or coiling at the internal carotid–AChA junction, endovascular treatment of arteriovenous malformations, and tumor resection along the optic tract or internal capsule [9]. Knowledge of AChA patency, caliber, and anatomical variants can help operators avoid inadvertent injury to this eloquent vessel and thereby reduce the risk of disabling complications. Furthermore, in vivo visualization of the AChA may facilitate the assessment of subtle flow compromise or vascular alterations in patients with chronic small-vessel disease or systemic vascular risk factors, which are not fully captured by parenchymal imaging alone [10].

Magnetic Resonance Angiography (MRA) has emerged as a significant non-invasive imaging technique that provides detailed images of blood vessels without radiation exposure. Time-of-Flight (TOF) MRA, a specific variant, is well-regarded for its high spatial resolution and signal-to-noise ratio (SNR), making it highly suitable for imaging complex cerebral vessels [11]. However, conventional MRA systems often struggle to accurately depict small and intricate vessels like the AChA. The inherent limitations in signal-to-noise ratio at these field strengths can result in incomplete visualization of distal segments and reduced diagnostic confidence, particularly for vessels with slow flow or complex geometry [12]. To address this, ultra-high-field (UHF) MR has been developed. A fundamental principle of MR physics is that a higher field strength yields a greater SNR, which can be leveraged for higher spatial resolution for visualizing smaller structures [13]. An important practical advantage of higher field strength is the ability to support smaller voxel acquisition while preserving adequate signal-to-noise ratio and clinically acceptable examination times. As noted in the review, UHF imaging significantly improves the delineation of microvasculature [14]. This technological advancement offers the potential to clearly visualize arteries as small as the AChA non-invasively.

Notably, recent advances in ultra-high-field TOF-MRA (5T–7T) have demonstrated improved visualization of intracranial vessels compared with standard 3T imaging [7]. Previous studies have explored the benefits of UHF-MRA for intracranial vascular imaging. Research comparing 3T and 7T TOF-MRA has consistently demonstrated that the higher field strength provides superior depiction of smaller vessels, including perforating arteries around the Willis ring [15]. A study by Shi et al. comparing 3T, 5T, and 7T confirmed that the conspicuity of distal small cerebral arteries progressively improves with increasing field strength [16]. Although previous studies have shown that ultra-high-field MRA improves conspicuity of small intracranial vessels, few studies have specifically evaluated the AChA. In addition, compared with 7T systems, 5.0T MRI may provide a more practical balance between increased SNR and reduced susceptibility-related limitations, potentially supporting high-resolution vascular imaging with greater clinical feasibility. Therefore, we designed this paired study to determine whether 5.0T provides measurable advantages for AChA visualization compared with 3.0T.

## Methods

### Study design and patients

This retrospective study was conducted at our hospital between May and August 2023 and included patients who underwent both 3.0T and 5.0T time-of-flight MR angiography (TOF-MRA) within a short interval. The inclusion criteria were patients who underwent both 3.0T and 5.0T TOF-MRA within 48 h for physical examination or evaluation of ischemic cerebrovascular symptoms. The order of 3.0T and 5.0T examinations was randomization by clinical workflow and scanner availability. The symptoms included headache, dizziness, hemisensory disturbance, and focal epilepsy.

Exclusion criteria included patients with: (1) a history of aneurysm surgery or intracranial aneurysm clip implantation; (2) cerebral hemorrhage or extensive infarction; (3) heart surgery or artificial heart valve implantation; (4) diabetes mellitus, which was excluded because of its known association with cerebral small vessel disease and microvascular alterations that could confound vascular visualization; (5) plans for pregnancy within 3 months or breastfeeding; and (6) claustrophobia.

This retrospective paired observational study was reported in accordance with the STROBE(Strengthening the Reporting of Observational Studies in Epidemiology) guidelines. This study was approved by the institutional ethics committee, and the requirement for informed consent was waived by the Institutional Review Board of our Hospital because of the retrospective nature of the study.

### Data collection and imaging analysis

#### Image acquisition and analysis

All images included in this retrospective study were acquired using either a 3.0T Philips Ingenia MR scanner with a 24-channel head coil or a 5.0T UIH Jupiter MR scanner with a 48-channel skull coil. The acquisition time for each TOF-MRA sequence was 3 min 16 s at 3.0T and 3 min 56s at 5.0T. Detailed TOF-MRA scanning parameters are available in Supplementary Table 1. All paired 3.0T and 5.0T examinations were completed within 48 h for each patient.

All image post-processing and measurements were performed on a FUJIFILM 3D SYNAPSE workstation and uOmnispace workstation using the vessel analysis tool, which was used to trace the vessel course and obtain length and diameter measurements. Two radiologists (with 8 and 10 years of experience), blinded to the MR field strength, independently evaluated all images, and the final value was calculated as the mean of the two observers’ results. Inter-rater reliability was strong (κ ≥ 0.7), and discrepancies in measurements or scores were resolved by joint re-evaluation and consensus.

#### Segmental classification of the AChA

For this study, the AChA was evaluated based on a microsurgical and angiographic four-segment classification scheme. The analysis focused on the first three segments which are visible on MRA, including A1 (The Origin segment), A2 (The Cisternal segment), and A3 (The Plexal segment). The fourth segment (Terminal/anastomotic) was not evaluated as it cannot be reliably resolved on routine MR imaging.

#### Subjective analysis

Subjective evaluation of vascular display quality: The clarity of the basilar artery (BA) and AChA segments (AChA-A1, AChA-A2, and AChA-A3) was evaluated using the three-subscale method on the Maximum Intensity Projection (MIP) and three-dimensional reconstruction maps of TOF-MRA. The scoring system is as follows: 3 points: The vascular segments are clearly displayed with uniform thickness and can be used for diagnosis; 2 points: The blood vessels are segmented and visible, but the signal contrast is poor, which does not affect the diagnosis; 1 point: The vascular segments are displayed blurry, affecting the diagnosis; 0 points: Vascular segments are not visible. A score of ≥ 2 indicates excellent vascular performance.

#### Objective analysis

**Fig. 1:**
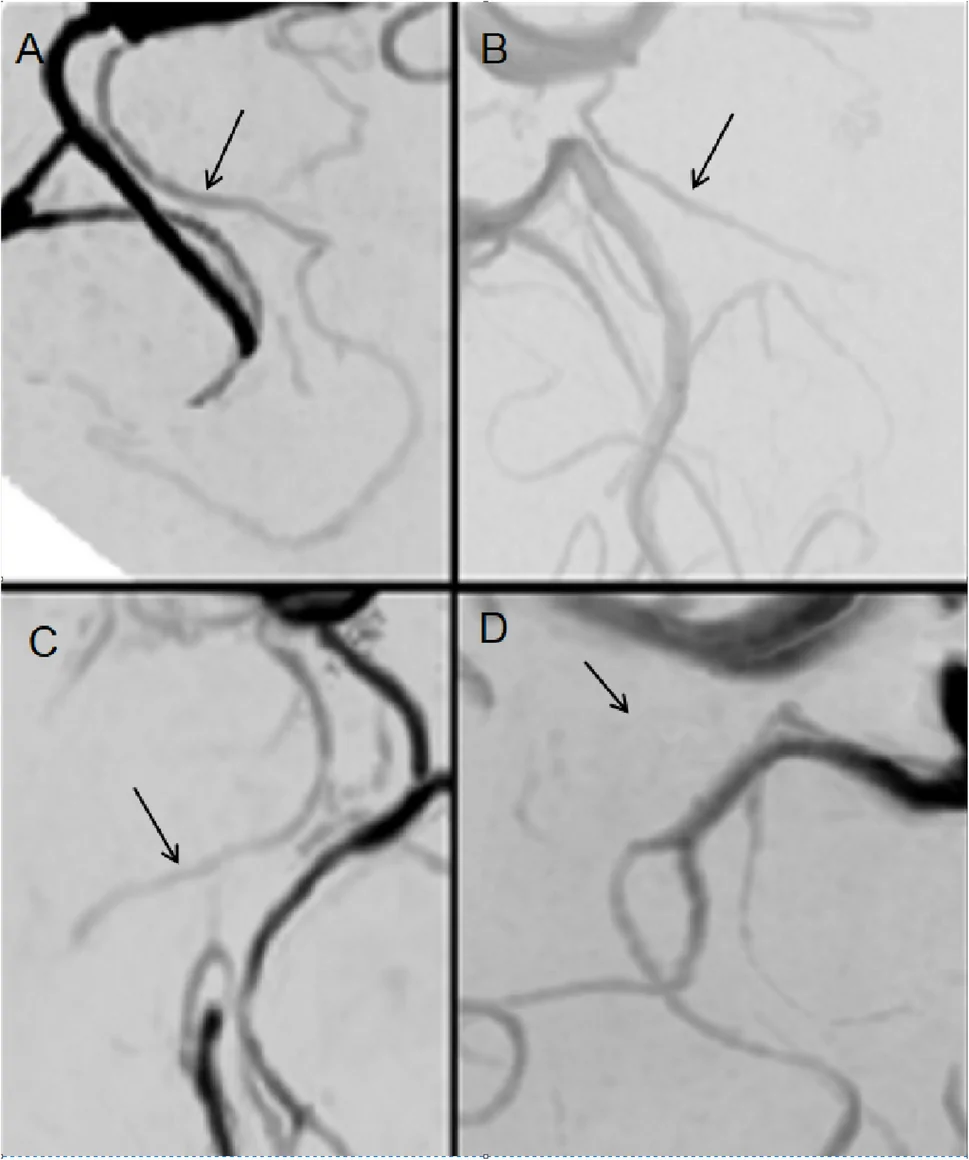
Representative examples of AChA visibility scores: grade 3, **A** clearly visualized vessel; grade 2, **B** visible but with reduced conspicuity; grade 1, **C** blurred visualization affecting diagnostic confidence; grade 0, **D** not visible

The quality of the images was determined using the original TOF-MRA images. For objective image quality assessment, a circular ROI was placed in the middle portion of the basilar artery, and a reference ROI was placed at the same level in the brainstem. The AChA length and diameter were measured using the vessel analysis tools of the workstation (Fig. 1), rather than manual ROI-based measurements. To reduce measurement bias related to voxel size differences, all 3.0T datasets were resampled to the 5.0T voxel grid before quantitative diameter measurement. SNR and Contrast-to-Noise Ratio (CNR) were calculated using the following formulas:

$$\mathrm{SNR} = (\text{Mean SI of the basilar artery} / \text{Standard deviation of SI in the brainstem}) \times 100\%$$

$$\mathrm{CNR} = (\text{Mean SI of the basilar artery} - \text{Mean SI of the brainstem}) / \text{Standard deviation of SI in the brainstem} \times 100\%$$

### Statistical analysis

Statistical analysis was performed using SPSS 26.0 software. The Shapiro-Wilk test was used to assess the normality of the data. The continuous variables that conformed to normal distribution were expressed as mean ± standard deviation for normally distributed data, and paired-sample t-tests were employed for comparison. Continuous variables with a non-normal distribution were expressed as median (Q1, Q3) and compared using the Wilcoxon signed-rank test. Categorical variables were summarized descriptively and were not subjected to between-group comparison due to the paired study design. Intra- and inter-observer consistency in image quality and vascular display was tested using the Intraclass Correlation Coefficient (ICC), with ICC > 0.75 considered good consistency. A two-sided *p*-value < 0.05 was considered as statistically significant.Paired-sample t-tests were used to compute mean differences and 95% CIs for continuous variables. For non-normally distributed data, the Wilcoxon signed-rank test was used, and results are presented as median (Q1, Q3) with z and *P* values.

## Results

### Patient selection and characteristics

A total of 42 patients who underwent both 3.0T and 5.0T TOF-MRA within 48 h were included in the paired analysis. The study cohort comprised 23 males (54.76%) and 19 females (45.24%), with a mean age of 59.34 ± 13.72 years. Vascular risk factors included smoking in 11 patients (26.19%), alcohol consumption in 16 patients (38.09%), hypertension in 18 patients (42.86%), hyperlipidemia in 12 patients (28.57%), cerebral small vessel disease in 18 patients (42.86%), and coronary heart disease in 7 patients (16.67%). Baseline clinical characteristics of the paired cohort are summarized in Table 1.

**Table 1:** Clinical characteristics of the study cohort

Parameter	No. of patients (*n* = 42)
Sex	
Male	23 (54.76)
Female	19 (45.24)
Age, years	59.34 ± 13.72
Smoking	11(26.19)
Drinking	16 (38.09)
Hypertension	18(42.86)
Hyperlipidemia	12(28.57)
Cerebral small vessel disease	18(42.86)

Values are presented as n (%), or mean ± standard deviation, as appropriate

### Evaluation of image quality

Quantitative parameters of bilateral AChA measured by 3.0T and 5.0T MRI are presented in Table 3. Both the major axis length and diameter of the left and right AChA were significantly larger at 5.0T than at 3.0T (all *P* < 0.01). Reliability analysis showed good to excellent inter-rater and intra-rater agreement, with ICC values ranging from 0.86 to 0.97, indicating high reproducibility of the measurement method at both field strengths. In the paired cohort, subjective visualization scores of the AChA segments demonstrated a stepwise improvement with increasing segmental distance on 5.0T TOF-MRA. Median AChA-A2 scores increased from 2.0 (Q1–Q3: 1.0–3.0) on 3.0T to 3.0 (3.0–3.0) on 5.0T (z = -3.51, *P* = 0.007), while AChA-A3 scores increased from 1.0 (0–2.0) to 3.0 (2.0–3.0), respectively (z = -5.37, *P* < 0.001). The overall AChA visualization score also showed a significant increase on 5.0T TOF-MRA (median 5.0 [4.0–5.0] vs. 5.0 [3.0–5.0]; z = -6.41, *P* < 0.001) (Table 2).

**Table 2:** Comparison of subjective AChA visualization scores and objective image quality metrics between 3.0T and 5.0T TOF-MRA in the paired cohort

	3.0T (*n* = 42)	5.0T (*n* = 42)	z/t	*p*
BA	3.0(3.0, 3.0)	3.0(3.0, 3.0)	0	1.000
AChA-A1	3.0(2.0, 3.0)	3.0(3.0, 3.0)	-0.76	0.329
AChA-A2	2.0(1.0, 3.0)	3.0(3.0, 3.0)	-3.51	0.007
AChA-A3	1.0(0, 2.0)	3.0(2.0, 3.0)	-5.37	< 0.001
Score, M (Q1, Q3)	5.0(3.0, 5.0)	5.0(4.0, 5.0)	-6.41	< 0.001
SNR (X̅± s)	65 ± 3	93 ± 5	-10.258	< 0.001
CNR (X̅± s)	52 ± 4	75 ± 6	-8.256	< 0.001

Values are presented as *n* (%), mean ± standard deviation, or median (Q1, Q3), as appropriate

Z values were derived from the Wilcoxon signed-rank test for paired comparisons. X̄ ± s denotes mean ± standard deviation. *t* values from paired *t*-test

*Abbreviations*: *AChA* Anterior choroidal artery, *BA* Basilar artery, *SNR* Signal-to-noise ratio, *CNR* Contrast-to-noise ratio

Objective image quality metrics further demonstrated significantly higher signal-to-noise ratio (SNR: 93 ± 5 vs. 65 ± 3; mean difference = 28, 95% CI: 24–32; *t* = -10.258, *P* < 0.001) and contrast-to-noise ratio (CNR: 75 ± 6 vs. 52 ± 4; mean difference = 23, 95% CI: 19–27; *t* = -8.256, *P* < 0.001) on 5.0T compared with 3.0T TOF-MRA. Distributional comparisons of these metrics are shown in Supplementary Fig. 1.

### Comparison of intracranial AChA length and diameter scores

Paired morphological measurements demonstrated significantly greater AChA length and diameter on 5.0T TOF-MRA compared with 3.0T imaging (Table 3). The mean length of the left AChA increased from 18.7 ± 9.2 mm at 3.0T to 24.4 ± 7.3 mm at 5.0T (mean difference = 5.7 mm, 95% CI: 3.8–7.6; t = 5.82, *P* < 0.001), while the right AChA length increased from 17.9 ± 8.4 mm to 23.3 ± 9.0 mm, respectively (mean difference = 5.4 mm, 95% CI: 1.9–8.9; t = 3.12, *P* = 0.003). The median diameter of the left AChA increased from 0.9 mm (0.7–1.1) at 3.0T to 1.1 mm (1.0-1.2) at 5.0T (z = -4.77, *P* < 0.001), and the right AChA diameter increased from 0.9 mm (0.8-1.0) to 1.1 mm (1.0-1.2) (z = -4.05, *P* < 0.001), indicating improved delineation of small-caliber vessels at higher field strength.

**Table 3:** Quantitative measurements and reliability analysis of AChA parameters between 3.0 T and 5.0 T MRI

Parameter	Field Strength	Mean ± SD (mm)	*z*/t	*P*-value	Inter-rater ICC (95% CI)	Intra-rater ICC (95% CI)
L AChA major axis	3.0T	18.7 ± 9.2*	5.82	< 0.001	0.93	0.97
	5.0T	24.4 ± 7.3*			0.95	0.96
R AChA major axis	3.0T	17.9 ± 8.4*	3.12	0.003	0.92	0.95
	5.0T	23.3 ± 9.0*			0.94	0.96
L AChA diameter	3.0T	0.9 (0.7–1.1)	-4.77	< 0.001	0.87	0.88
	5.0T	1.1 (1.0-1.2)			0.88	0.88
R AChA diameter	3.0T	0.9 (0.8-1.0)	-4.05	< 0.001	0.86	0.87
	5.0T	1.1 (1.0-1.2)			0.87	0.88

ICC, intraclass correlation coefficient. Inter-rater ICC: agreement between two independent observers; intra-rater ICC: repeated measurement consistency of a single observer

*Data expressed as mean ± SD for normally distributed data and median (IQR) for non-normally distributed data. Paired *t*-test and Wilcoxon signed-rank test were used for inter-field strength comparison

**Fig. 2:**
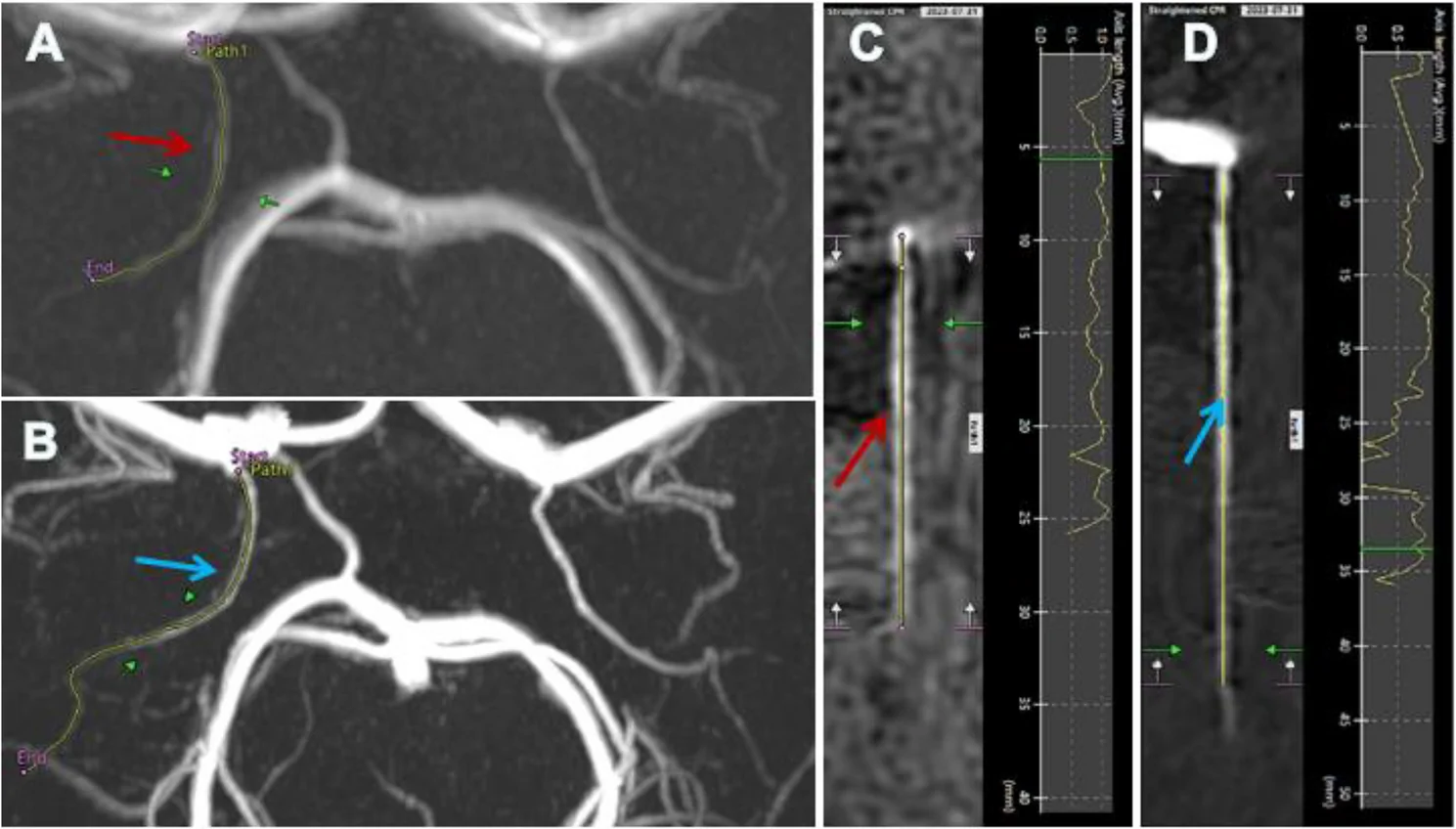
Comparative MIP images of the AChA for the same subject on 3.0T (**A** and **C**) and 5.0T (**B** and **D**)

**Fig. 3:**
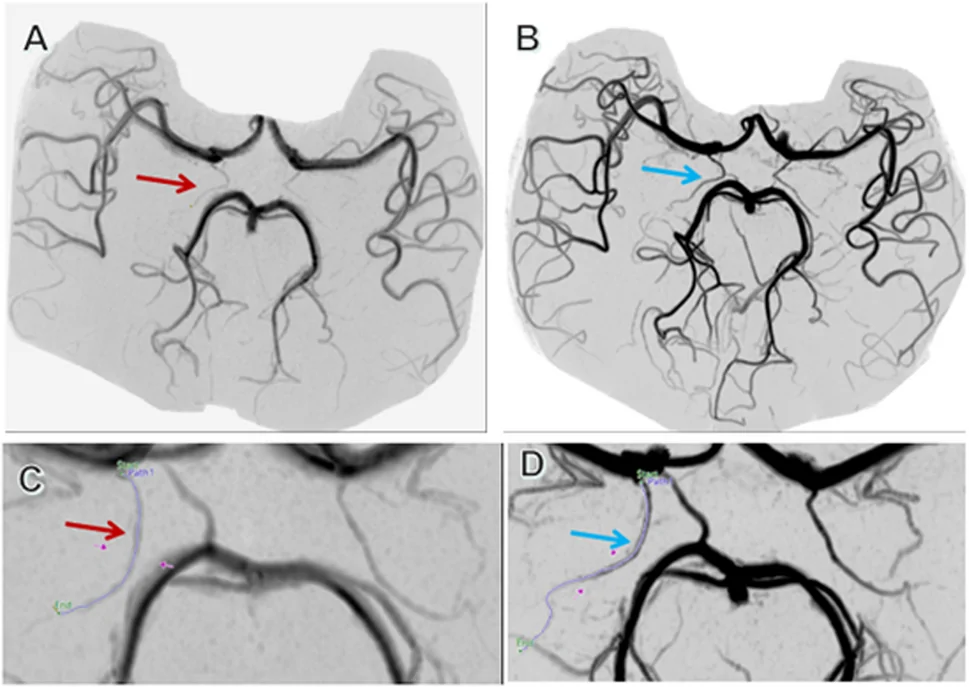
Reversed color brain MRA images from the same subject, comparing 3.0T (**A** and **C**) and 5.0T (**B** and **D**) MR scans. Red arrows indicate the anterior choroidal artery (AChA) visualized on 3.0T images, while blue arrows highlight the AChA on 5.0T images

**Fig. 4:**
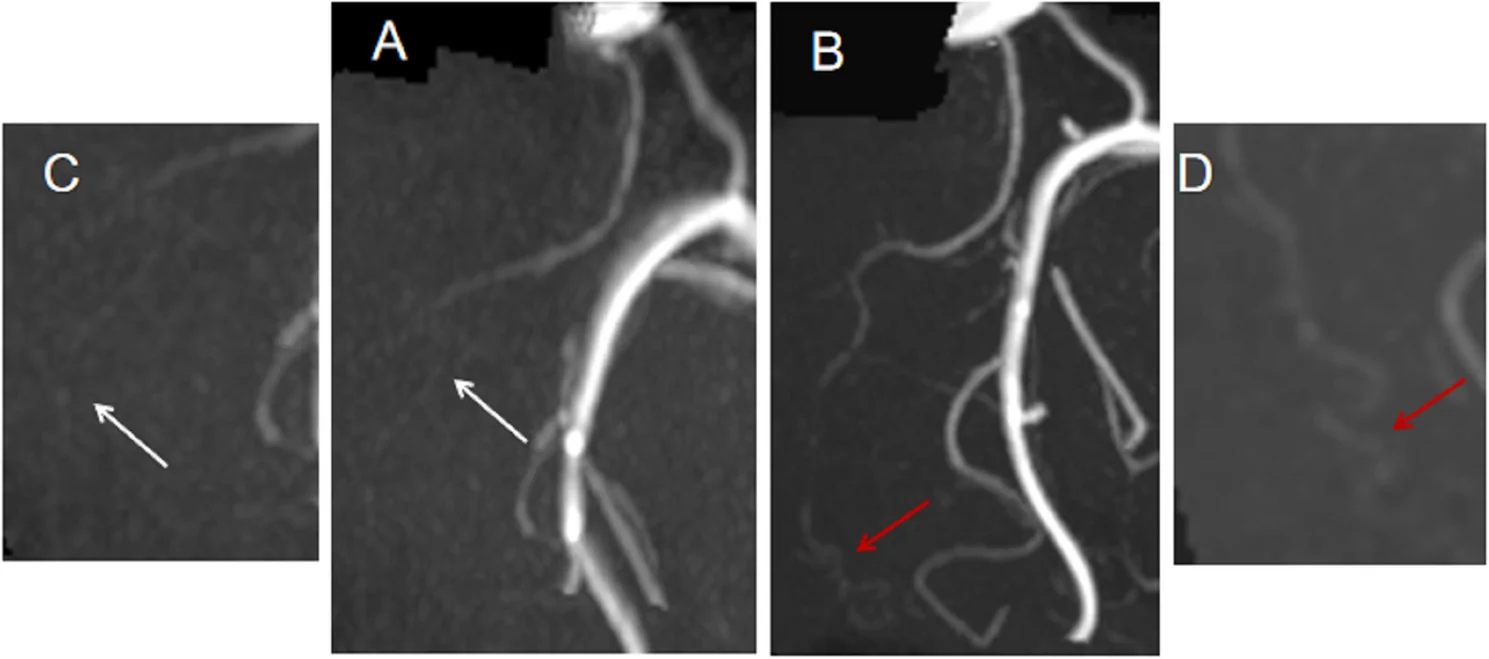
Comparison of the distal segments of the anterior choroidal artery (AChA) in the same subject on 3.0T (**A** and **C**) and 5.0T (**B** and **D**)

Representative paired measurements are shown in Fig. 2, with corresponding reversed-color MRA images in Fig. 3. Magnified distal vascular segments are presented in Fig. 4. A representative diseased case with moyamoya disease, illustrating improved conspicuity of the AChA on 5.0T compared with 3.0T TOF-MRA, is provided in Supplementary Fig. 2.

## Discussion

In this paired retrospective analysis, 5.0T TOF-MRA demonstrated improved objective and subjective image quality compared with 3.0T imaging for visualization of the anterior choroidal artery. Improvements were most pronounced in distal AChA segments (A2 and A3), with higher SNR, CNR, and visualization scores. These findings indicate that higher field-strength TOF-MRA enhances visualization of small-caliber intracranial arteries.

These findings are consistent with prior studies reporting that ultra-high-field MRI provides enhanced signal and spatial resolution for imaging small-caliber intracranial vessels [7, 17]. Specifically, the 5.0T system exhibited a higher detection rate of AChA segments, superior SNR, and CNR, consistent with previous studies conducted at 7.0T and 5.0T field strengths [7].

In previous comparative studies, 7.0T TOF-MRA has been reported to enhance visualization of small intracranial arteries, including the lenticulostriate arteries and anterior perforators, due to the inherent increase in SNR that accompanies stronger magnetic fields [7, 18]. However, limitations of 7.0T, including increased susceptibility artifacts and B1 inhomogeneity, have hindered its widespread clinical use [19]. Compared with 7.0T, 5.0T may offer a more practical balance between improved signal performance and clinical feasibility. Although the 5.0T protocol employed smaller voxel dimensions than the 3.0T acquisition, this difference reflects an inherent advantage of higher field-strength imaging. The increased intrinsic SNR at 5.0T permits high-spatial-resolution TOF-MRA to be performed within clinically acceptable scan durations. Achieving a comparable voxel size at 3.0T would likely require longer acquisition times or compromise image quality because of reduced SNR efficiency. Therefore, the observed improvement at 5.0T likely reflects the combined benefit of higher field strength and the practical feasibility of high-resolution acquisition. The present results suggest that 5.0T offers meaningful improvements in vessel conspicuity and quantitative image quality while avoiding several practical limitations associated with 3.0T and 7.0T imaging. Beyond TOF angiography, the advantages of higher field strength also extend to vessel wall imaging [20, 21].

Subjective image analysis in this study further indicated that 5.0T TOF-MRA offers superior clinical utility. The depiction of the AChA, especially in its distal segments (A2 and A3), was significantly clearer on 5.0T images. This is of particular clinical relevance, as the distal portions of the AChA supply eloquent brain regions [2] and are often not well resolved with 3.0T imaging. Enhanced visibility of such small perforating arteries at higher field strength contributes to more accurate preoperative risk stratification and surgical planning [22, 23]. In neurosurgical and endovascular procedures, inadvertent AChA occlusion has been reported in approximately 2–5% of cases, often leading to severe neurological deficits such as hemiplegia or hemianopsia [24, 25], highlighting the clinical value of precise AChA visualization to minimize procedural complications. Specifically in cerebrovascular disease management, ultra-high-field MRA has been shown to be invaluable for identifying perforator arteries adjacent to aneurysms or arteriovenous malformations, directly influencing treatment strategies to minimize ischemic complications [26]. The subjective grading scores in the present study, corroborated by high inter-rater agreement, indicate that 5.0T imaging may provide increased diagnostic confidence, especially when evaluating small vessels that are critical yet vulnerable during neurovascular interventions, which may support more confident anatomical assessment during preoperative planning. This is consistent with the general principle in MRA that improved SNR and CNR directly translate to better vessel conspicuity and fewer artifacts, which are key determinants of a radiologist’s diagnostic confidence and accuracy [27, 28].

Secondary measurements, including vessel length and diameter, also revealed statistically significant differences between 5.0T and 3.0T imaging. The observed greater vessel length and diameter at 5.0T are indeed a reflection of improved distal segment detection. A likely contributing factor is the reduced influence of partial volume effects at higher spatial resolution [29, 30]. At 3.0T’s lower spatial resolution, the signal from a fine vessel like the AChA is averaged with surrounding non-vascular tissue within a single voxel, causing the vessel to appear artificially smaller and its distal segments to be obscured [31]. The superior resolution of 5.0T minimizes this effect, allowing for a more accurate delineation of the vessel’s true anatomical boundaries and dimensions [32, 33]. High inter-observer consistency across these metrics further supports the robustness of the imaging protocol and the reliability of the findings.

The improved depiction of small distal intracranial arteries at 5.0T may have potential clinical applications in aneurysm planning, perforator artery assessment, and the evaluation of moyamoya disease, where precise visualization of small-caliber vessels is important. However, prospective studies are needed to determine whether these imaging advantages translate into improved diagnostic or procedural outcomes.

Several limitations of this study should be acknowledged. First, this was a retrospective, single-center analysis, which may limit generalizability. Second, although a paired design was used to minimize intersubject variability, the 3.0T and 5.0T examinations were performed using different hardware configurations, including head coils with different channel counts. The 5.0T system employed a higher-channel head coil, which is known to improve signal reception efficiency, parallel imaging performance, and overall signal-to-noise ratio, particularly for small-caliber and peripheral vessels [34]. Differences in coil geometry, element density, and radiofrequency sensitivity profiles may therefore have contributed to the observed improvements in SNR, CNR, and distal AChA visualization, independent of field strength alone. As a result, the superior image quality observed at 5.0T likely reflects the combined effects of increased magnetic field strength and optimized hardware design rather than field strength in isolation. Importantly, the smaller voxel size achievable at 5.0T should be interpreted as part of the field-strength advantage, because comparable spatial resolution at 3.0T would likely require substantially longer acquisition times or reduced image quality. Future studies using standardized coil configurations or cross-system normalization strategies would help further disentangle the relative contributions of field strength and hardware to small-vessel MRA performance. In addition, the present study did not include dedicated vessel sharpness or edge-definition analysis. Although the observed increases in distal vessel conspicuity, measurable vessel length, and measurable vessel diameter indirectly support improved delineation of small-caliber vessels at 5.0T, future studies incorporating quantitative vessel sharpness metrics or point-spread-function-based evaluation may help further clarify the relative contributions of field strength, spatial resolution, and reduced partial-volume averaging to small-vessel depiction. Finally, because the AChA supplies eloquent deep structures, improved delineation of its course may be useful for preoperative planning and anatomic risk assessment in aneurysm, AVM, or tumor procedures. However, the present study was designed to evaluate image quality rather than clinical outcomes, so the direct impact on management remains to be established. Prospective studies with standardized hardware and outcome-based endpoints are warranted to further clarify the clinical implications of higher field-strength TOF-MRA.

## Conclusions

In a paired cohort, 5.0T TOF-MRA demonstrated improved objective and subjective image quality compared with 3.0T imaging for visualization of the anterior choroidal artery, particularly its distal segments. The higher intrinsic SNR at 5.0T enabled high-resolution imaging within clinically feasible acquisition times, contributing to improved depiction of small-caliber intracranial vessels. Further studies are required to determine its clinical implications.

## Supplementary Information

Supplementary Material 1.

Supplementary Material 2.

Supplementary Material 3.

## Data Availability

All data generated or analysed during this study are included in this published article.
