# Supplementary material for: Comparison of 5.0T and 3.0T time-of-flight MR angiography for visualizing the anterior choroidal artery
Source: BMC Neurol. 2026 Jun 12;26:529. doi: 10.1186/s12883-026-05019-9 (PMC13495236; doi:10.1186/s12883-026-05019-9)
Supplement: Supplementary file 1 [file 12883_2026_5019_MOESM3_ESM.docx]

**Supplementary Figure 1.** Paired quantitative comparison between 3.0T and 5.0T TOF-MRA.

A) Box plot of the anterior choroidal artery (AChA) major axis length measured on 3.0T and 5.0T TOF-MRA.

B) Box plot of the AChA diameter measured on 3.0T and 5.0T TOF-MRA.

C) Box plot of the signal-to-noise ratio (SNR) measured on 3.0T and 5.0T TOF-MRA.

D) Box plot of the contrast-to-noise ratio (CNR) measured on 3.0T and 5.0T TOF-MRA.

**Supplementary Figure 2.** Representative Time-of-Flight MR angiography images in a patient with moyamoya disease.

A) 3.0T TOF-MRA showing limited visualization of the anterior choroidal artery (AChA) in the setting of moyamoya disease.

B) 5.0T TOF-MRA of the same patient demonstrating improved visualization of the AChA and distal arterial segments.

| Field strength (B_0_) | TR (ms) | Voxel size (mm^3^) | TE (ms) | FA (°) | FOV (mm^2^) | Scanning time |
| --- | --- | --- | --- | --- | --- | --- |
| 3T | 20 | 0.63×0.63×0.63 | 3.5 | 15 | 224×181 | 3 min 16 s |
| 5T | 20 | 0.5×0.5×0.5 | 3.8 | 20 | 224×180 | 3 min 56s |

**Supplementary Table 1. Acquisition parameters for MRA at 3T and 5T**
